# Rediscovery of one of the world's rarest sharks, the sailback houndshark *Gogolia filewoodi* , in Papua New Guinea

**DOI:** 10.1111/jfb.70196

**Published:** 2025-08-21

**Authors:** Jack Sagumai, Rebecca H. Samuel, William T. White, Michael I. Grant

**Affiliations:** ^1^ WWF Pacific, PNG Marine Programme Madang Papua New Guinea; ^2^ CSIRO Australian National Fish Collection Hobart Tasmania Australia; ^3^ Centre for Sustainable Tropical Fisheries and Aquaculture, College of Science and Engineering James Cook University Townsville Queensland Australia; ^4^ Piku Biodiversity Network, National Research Institute Port Moresby Papua New Guinea

**Keywords:** Astrolabe Bay, endemic, fishers, Gogol River, Madang, Triakidae

## Abstract

A recent survey of artisanal and subsistence fishers in Madang, Papua New Guinea, resulted in rediscovery of the sailback houndshark *Gogolia filewoodi*. The five females and one male *G. filewoodi* recorded in 2020 and 2022 near the Gogol River mouth are the first verified records of this species since its description from a single specimen in the 1970s. *Gogolia* is a monotypic triakid genus and thus represents a unique evolutionary lineage not seen anywhere else in the world and could be an important marine biodiversity icon for Papua New Guinea.

The sailback houndshark *Gogolia filewoodi* Compagno, 1973 was described based on a pregnant female containing two late‐term embryos. The holotype was collected in 1970 by L.W. Filewood near the Gogol River mouth in Astrolabe Bay, Madang, in northern Papua New Guinea (Compagno, 1973; White & Ko'ou, 2018). Despite a dedicated study of PNG sharks and rays conducted between 2013 and 2017, no new records of this species were found (White & Ko'ou, 2018). Previous deep‐sea surveys for marine fauna conducted from 2010 to 2014 in PNG, including in Madang, did not record this species, although these surveys did identify six new species of sharks and rays (Fricke et al., 2014; White et al., 2017; White & Ko'ou, 2018). *G. filewoodi* is currently listed as data deficient by the International Union for Conservation of Nature (IUCN) Red List of Threatened Species as a lack of records preclude any understanding of population size or trend (Sherman et al., 2020). Creel and market surveys in the Madang Province conducted by the World Wildlife Fund (WWF) in Papua New Guinea between February and May 2020 recorded five females of this species in the same geographic area where the type specimen was collected in 1970 (Compagno, 1973; WWF, 2020). These individuals possess the characteristic long first dorsal‐fin base, which is unique among the triakid sharks.

The five *G. filewoodi* individuals were caught near the Gogol River mouth at a depth of 80 m by a fisher from the Bilbil village who was targeting jewfish (Sciaenidae) using handlines (Figure 1a,b). Two individuals measuring 61.0 and 60.0 cm in stretched total length (*L*_T_) were caught on 18 March 2020, and three individuals measuring 75.5, 76.1 and 59.0 cm *L*_T_ were caught on 19 March 2020. All individuals were adult females, except for the 59.0‐cm *L*_T_ female, which was adolescent. The catch data were recorded by a community facilitator for the WWF in Bilbil village conducting fisheries surveys at the time. He recorded the data of the first two catches without taking photographs but did take photographs of the other three individuals as evidence of the species he was recording. One of the individuals recorded contained five well‐developed oocytes in the functional ovary. In September 2022, a 73‐cm *L*_T_ adult male *G. filewoodi* was recorded, which had been caught by a fisher from Bilbil village (Figure 1c). This individual was also caught close to the Gogol River mouth at a depth of ~200 m near Umin village. This individual is the first male recorded for *G. filewoodi*. No experiment on live or recently captured specimens was conducted in this study; specimens examined were already deceased as fisheries catch.

**FIGURE 1:**
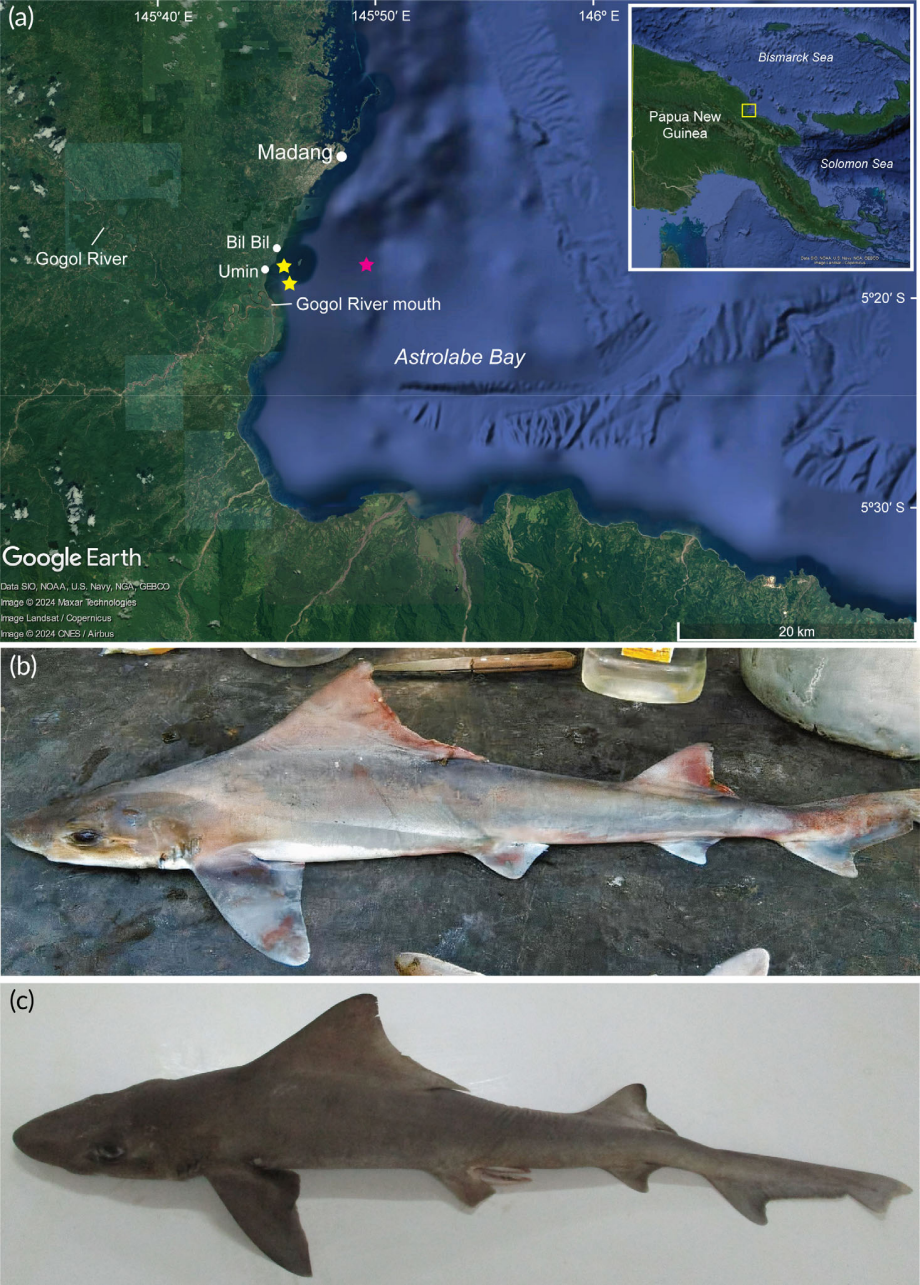
(a) Map of Papua New Guinea showing the Astrolabe Bay area in the yellow box (inset). Yellow stars denote the locations where fishers caught *Gogolia filewoodi* specimens, and the pink star denotes holotype collection location. (b) Adult female [76.1 cm total length (*L*_T_)] and (c) adult male (73 cm *L*_T_) specimens of *G. filewoodi* caught near the mouth of the Gogol River.

Anecdotal reports from fishers in Bilbil village and within Madang Lagoon indicate that the species is caught occasionally when fishing within Astrolabe Bay. Despite their fishing activity extending throughout Astrolabe Bay and adjacent coast, all observed interactions with *G. filewoodi* occurred in Astrolabe Bay, close to the Gogol River mouth. Anecdotal reports suggest that *G. filewoodi* is caught as by‐catch by fishers targeting jewfish (Sciaenidae) for the fish maw (teleost swim bladder) trade during the months of March–July and during the dry season from August to November. The present observations of six specimens of *G. filewoodi* were all caught between March 2020 and September 2022, when fishers target jewfish near the Gogol River mouth. Fishers report that its flesh is not well regarded and often given away if in surplus (Don Giling, personal communication), and the fins are not high quality for the shark‐fin trade. *G. filewoodi* is thought to be a continental shelf species, mainly occurring in deeper waters (White & Ko'ou, 2018). The continental shelf off northern mainland PNG only stretches a few kilometres off the coast, and this species appears to interact with fishers in deeper trenches that extend inshore to Astrolabe Bay and the Gogal River mouth. Despite comprehensive marine fauna surveys within PNG waters and within Madang, the species has not been discovered elsewhere. The new records documented during the WWF market surveys were close to the type locality for the species, suggesting that the geographic range of this species is possibly restricted to a limited region around Astrolabe Bay. This possible micro‐endemism could make this species susceptible to population declines from increased fishing effort in the future. This study also highlights the importance of artisanal surveys in poorly studied regions.

Overfishing and habitat loss are the key drivers of population declines of sharks and rays, and there are increasing concerns about their conservation and management worldwide (Dulvy et al., 2021). Considering its rare occurrence and possible micro‐endemism in Astrolabe Bay in the vicinity of the Gogol River mouth, the catch of five individuals over two survey days suggests that it could be a common by‐catch species of the fish maw fishery at least near the Gogol River mouth. Targeted fish maw fisheries are increasing throughout regional PNG and have been identified as a major pressure to threatened species of sawfish (Pristidae), river sharks (*Glyphis* spp.) and winghead sharks (*Eusphyra blochii*) in southern PNG (Amepou et al., 2024; Grant et al., 2021, 2022). In Astrolabe Bay, growing interest in the fish maw trade could be an imminent threat to this endemic species, and further artisanal surveys are required. Rare and endemic species are vulnerable to extinction due to their limited distribution range and increasing anthropogenic threats in their habitats (Isik, 2011). Despite the present scientific rediscovery, fundamental information is still lacking on its ecology, life history and distribution. Monitoring and management options are currently being initiated as a precautionary approach to conserve this unique and rare species of shark *G. filewoodi* represents a unique evolutionary lineage of triakid sharks and could be considered an important marine biodiversity icon for Papua New Guinea.

## AUTHOR CONTRIBUTIONS

Ideas: Jack Sagumai, Rebecca H. Samuel, William T. White and Michael I. Grant. Data generation and analysis: Jack Sagumai. Manuscript preparation: Jack Sagumai, William T. White and Michael I. Grant. Funding: WWF Hong Kong.
